# Interprofessional actions in responsible discharge: contributions to transition and continuity of care

**DOI:** 10.1590/1980-220X-REEUSP-2022-0452en

**Published:** 2023-12-04

**Authors:** Tatiane Cristina Zanetoni, Danielle Fabiana Cucolo, Marcia Galan Perroca

**Affiliations:** 1 Faculdade de Medicina de São José do Rio Preto, Programa de Pós-Graduação em Enfermagem, São José do Rio Preto, SP, Brazil.; 2 Pontifícia Universidade Católica de Campinas, Programa de Residência Multiprofissional em Saúde, Campinas, SP, Brazil.

**Keywords:** Patient Discharge, Continuity of Patient Care, Patient Care Team, Transitional Care, Patient-Centered Care, Alta del paciente, Continuidad de la Atención al Paciente, Grupo de Atención al Paciente, Cuidado de Transición, Atención centrada en el Paciente, Alta do Paciente, Continuidade da Assistência ao Paciente, Equipe de Assistência ao Paciente, Cuidado Transicional, Assistência Centrada no Paciente

## Abstract

**Objective:**

To analyze the interprofessional team’s perception of the actions carried out by means of responsible hospital discharge, and their contribution to improving the transition and continuity of patient care.

**Methods:**

A qualitative study was carried out in two hospitalization units, in October – November 2020, interviewing health professionals from a teaching hospital in the state of São Paulo. The reports were transcribed and subjected to thematic content analysis.

**Results:**

Twelve professionals participated (doctor, nurse, physiotherapist, nutritionist, speech therapist and social worker) and three thematic categories emerged from the interviews: 1. informational continuity in responsible discharge; 2. interaction between professionals and services for the transition of care; and 3. workload management for better transition and continuity of care.

**Conclusion:**

The team recognized interprofessional advances and challenges in responsible discharge related to the informational and relational continuity of patient care and highlighted the (over)workload as an unfavorable aspect in the transition process, generating impacts for patients, professionals and health services.

## RESUMO

**Objetivo:**

Analisar a percepção da equipe interprofissional sobre as ações realizadas na alta hospitalar responsável e sua contribuição para a melhoria da transição e continuidade do cuidado ao paciente.

**Métodos:**

Estudo qualitativo realizado em duas unidades de internação, em outubro e novembro de 2020, utilizando entrevista com profissionais de saúde de um hospital de ensino do interior do estado de São Paulo. Os relatos foram transcritos e submetidos à análise de conteúdo na modalidade temática.

**Resultados:**

Participaram 12 profissionais (médico, enfermeiro, fisioterapeuta, nutricionista, fonoaudiólogo e assistente social) e três categorias temáticas emergiram das entrevistas: 1. Continuidade informacional na alta responsável; 2. Interação entre os profissionais e serviços para a transição do cuidado; e 3. Gestão da carga de trabalho para melhor transição e continuidade do cuidado.

**Conclusão:**

A equipe reconheceu avanços e desafios interprofissionais na alta responsável relativos à continuidade informacional e relacional do cuidado ao paciente e destacou a (sobre)carga de trabalho como aspecto desfavorável no processo de transição gerando impactos para os pacientes, profissionais e serviços de saúde.

## RESUMEN

**Objetivo:**

Analizar la percepción del equipo interprofesional sobre las acciones llevadas a cabo en el alta hospitalaria responsable y su contribución a la mejora de la transición y continuidad de la atención de los pacientes.

**Método:**

Estudio cualitativo realizado en dos unidades de internación, en octubre y noviembre de 2020, mediante entrevistas a profesionales de salud de un hospital escuela del interior del estado de São Paulo. Los relatos fueron transcritos y sometidos a análisis de contenido temático.

**Resultados:**

Participaron 12 profesionales (médico, enfermera, fisioterapeuta, nutricionista, fonoaudióloga y asistente social) y de las entrevistas surgieron tres categorías temáticas: 1. continuidad informativa en el alta responsable; 2. interacción entre profesionales y servicios para la transición de los cuidados; y 3. gestión de la carga de trabajo para una mejor transición y continuidad de la atención.

**Conclusión:**

El equipo reconoció avances y retos interprofesionales en el alta responsable relacionados con la continuidad informativa y relacional de la atención al paciente y destacó la (sobre)carga de trabajo como aspecto desfavorable en el proceso de transición, generando impactos para pacientes, profesionales y servicios de salud.

## INTRODUCTION

Interprofessional collaboration calls for joint action between two or more members of the healthcare team, having a positive impact on the care provided, adherence to best practices and the use of resources^(1)^.

The development of a common therapeutic project, a dialogical relationship, recognition of the different types of knowledge and the participation of the patient/family in the care are attributes that make it possible to differentiate the effectiveness of teamwork^(2)^. Thus, the interdependence of practices allows to coordinate actions and plan collaboratively for the patient’s discharge, which makes the journey of care less stressful and safer^(3)^.

Despite the emphasis on the role of nurses^(4,5)^, other professionals in the healthcare team play an important role in the discharge process, identifying eligibility criteria, adopting educational programs and accompanying patients during their hospitalization and after they leave the institution^(6)^. When drawing up guidelines for de-hospitalization, transition and continuity of care in the Health Care Network (RAS in the Portuguese acronym), the Brazilian health system proposed the term “responsible discharge”. It highlights attributes such as patient/family autonomy, coordination between the hospital network, primary care and other levels of care, as well as interprofessional actions centered on the patient/family^(7)^.

Nurses^(4)^, doctors^(8)^, physiotherapists^(9)^ and social workers^(10)^ support the importance of communication and interprofessionalism, as well as networking, for the discharge process. This is important because continuity of health care is achieved through interpersonal relationships, the sharing of information and the effective coordination of interventions, being these elements a needed part of transitional care^(11)^. Otherwise, poor communication, the absence of common objectives and the lack of coordination of actions compromise the process^(8)^.

Continuity of care is discussed worldwide as a representation of the patient’s experience of healthcare and the interconnection of these elements in the care journey, aligned with their needs and preferences^(12)^.There are studies associating transition actions and continuity of care with a reduction in unplanned readmissions^(13)^ and lower disease complications, mortality and costs^(14)^. It is therefore important to understand how these interventions adapt to different healthcare settings and which approaches are most effective.

The majority of studies highlight the work and problems arising from the continuity of care (or lack of thereof) by focusing on individual categories, such as nurses^(4)^, doctors^(8)^ and social workers^(10)^ or physiotherapists^(9)^. Nationally, interprofessional work (IPW)^(7)^ and the transition of care in the de-hospitalization of patients have been gaining prominence^(5)^. However, the operationalization of responsible hospital discharge based on individual, collective and environmental forces^(15)^, proposed in this manuscript, has been little explored in the literature. In de-hospitalization, it is essential that professionals work both with the patient’s needs and potential and with family structuring and organization, with the support of the RAS^(5)^ and with interprofessional collaboration among services^(16)^.

This research is part of a master’s dissertation dealing with nursing time and the effectiveness of care transitions and is linked to the Health and Nursing Services Management research group (GESTSAÚDE in the Portuguese acronym). Its aim is to analyze the interprofessional team’s perception of the actions carried out during responsible hospital discharge and their contribution to improving the transition and continuity of patient care. Thus, it seeks to answer the following question: In the perception of the interprofessional team, what actions are carried out during the responsible discharge process that contribute to improving the transition and continuity of patient care?

## METHOD

### Type of Study

This is a descriptive, exploratory, qualitative study applying semi-structured interviews and based on the theoretical framework of Strengths-Based Nursing Care^(15)^. This theory advocates patient/family-centered care, aimed at developing autonomy and self-care, through collaborative practice and health promotion and prevention actions. It identifies individual and external competencies, capacities and resources as strengths^(15)^. The Consolidated criteria for reporting qualitative research (COREQ) tool^(17)^ was used to guide the design and presentation of the research.

### Study Site

The study was developed in two inpatient units, one medical-surgical and the other for palliative care, of a teaching hospital of special size, a reference in high complexity, located in a city of the state of São Paulo. Together, the units under study had 86 beds, with patients from different specialties requiring attention regarding the use of invasive devices (naso-enteral tube, delayed bladder catheter, gastrostomy, among others) and post-discharge care.

The responsible discharge process was established at the institution in November 2015. In the model adopted, healthcare professionals work concurrently in patient care and are also trained to carry out discharge planning, which should begin within the first few hours of the patient’s admission. The following eligibility criteria are taken into account: patients hospitalized with chronic diseases, in palliative care, using devices such as probes, catheters and stomas or health equipment, high-risk pregnant women, puerperal women with any comorbidity and premature newborns.

This process begins when the nurse identifies the frailty or eligibility criteria defined by the institution and then calls in the other members of the healthcare team to draw up the therapeutic project and discharge plan in an interprofessional manner. Throughout their hospitalization, patients must participate in care activities, receive guidance and be encouraged to make decisions about their treatment. The bed management service makes the connection with the RAS and thus monitors the process for drawing up the referral and counter-referral guide containing the post-discharge care proposal.

### Participants

All 15 health professionals, including doctors, nurses, physiotherapists, nutritionists, speech therapists and social workers, who were members of the care team and also responsible for discharge planning in the units investigated, were invited to participate. In order to take part in the study, they had to meet the following criteria: work during the daytime and have at least one year’s experience in the responsible discharge process; those who were on vacation and/or on different types of leave were excluded.

### Data Collection

Health professionals were contacted in person and by message via telephone application. After consulting on the best strategy for carrying out the interviews, due to the COVID-19 pandemic period, the decision was made to meet in person, in a reserved and easily accessible place within the institution. Security measures were duly complied with and data collection took place between October and November 2020.

One of the researchers with experience in the discharge process conducted the sessions that were held solely with the researcher and the invited professional. First, clarifications were made about the study and, after consenting, the participants answered sociodemographic questions (gender, age, profession, specifics about training, time at the institution and experience in responsible discharge).

The participants were then asked: 1) *How do you perceive the structuring/organization of the responsible discharge process in this institution in relation to the favorable aspects (for patients, professionals and the institution); and those that could be improved? 2) Is there integration between professionals and coordination between actions? 3) Is the time you dedicate to each patient sufficient/adequate? 4) Does the responsible/qualified discharge process effectively prepare the patient for continuing care at home*?

Data saturation was used as a parameter to determine the number of answers to the proposed questions^(18)^. It was reached when the twelfth interview was completed with two professionals from each area of knowledge.

The reports were recorded and the interviewer also recorded aspects to be clarified during or at the end of each meeting. Only one interview was carried out per professional, in the morning or afternoon, lasting approximately 20 minutes. The transcriptions were made in a Microsoft Word document, keeping the words in their entirety.

### Data Analysis and Processing

The study adopted the thematic content analysis^(19)^, without the aid of qualitative data management software, following the stages: pre-analysis, exploration of the material and treatment of the results. In the pre-analysis, the material was organized by grouping the professionals’ answers according to the interview questions: structuring and organization of the process; collaboration in the transition of care; time dedicated and preparation of the patient to continue post-discharge care.

The analysis began with a floating reading of the material and the organization of main points to formulate hypotheses based on the assumptions of continuous care^(11)^ and based on strengths^(15)^. As an exploration procedure, the material was coded into recording units and the deductive process was used to identify converging variables. In order to qualify the findings, the codes and themes were independently delineated by two researchers and then validated by a third.

To process and interpret the results, we proceeded with synthesis, data selection and inferences to extract the thematic categories agreed upon by the researchers and based on the theoretical framework^(16)^ and the literature on interprofessional work^(1,2)^, discharge planning, transition and continuity of patient care^(5,7,11,12,16)^.

### Ethical Aspects

The study is compliant with Resolution 466/2012 and was cleared by the Research Ethics Committee of the study institution under opinion number 4.347.495/2020. Data collection was carried out after the study objectives had been explained to the participants, and acceptance was obtained by signing the Free and Informed Consent Form.

In order to preserve anonymity, the participants were randomly identified with letters and numbers (E1, E2, E3,...E12) and professional category (Nurse, Doctor,...).

## RESULTS

The study included 12 female professionals, two from each professional category (doctors, nurses, physiotherapists, nutritionists, speech therapists and social workers), with an average age of 37.7 (SD = 8.2; range 27–47) years. They had been working at the institution for an average of 9.5 (SD = 7.5; range 1–24) years; eleven of them had a specialization course in various areas and the average length of experience in the responsible discharge process was four (SD = 1.9; range 1–7) years.

We found that nine codes emerged from the analysis of the reports: early discharge planning, patient/family participation in discharge, guidelines for continuing care, repercussions of post-discharge actions, communication between professionals (via WhatsApp, in team meetings and multi-professional visits), nurse mediator, (dis)articulation between services, dedicated time, telephone contact and post-discharge home visits. Three thematic categories were then extracted: Informational continuity in responsible discharge; Interaction between professionals and services for the transition of care and Workload management for better transition and continuity of care, the first of which was made up of the subcategories “Communication between professionals”, “Guidance for the patient/family/caregiver” and “Results perceived by the team”.

### Continuity of Information During Responsible Discharge

In order to plan hospital discharge and start transition actions, according to the interviewees, early communication from the medical team would favor the actions of the other professionals in the process. Although there were moments of interaction between the professionals, the decision on discharge proved to be centred on the doctor and the information shared did not occur in a timely manner or was not sufficient for the organization and qualification of the responsible discharge.

*(...) often the team calls it a qualified discharge, but in practice it doesn’t happen... the qualified discharge patient leaves... and there wasn’t a program. (E8 – Nutritionist)*

*I think the medical team could give more advance notice. (E11 – Nurse)*

*I think that communication between the team could be improved, because it’s not every day that there’s a visit (...) so sometimes there’s a lack of organization and communication. (E2 – Physiotherapist)*

They also highlighted moments of inclusion of the patient/family/caregiver in clinical decisions and guidelines for the development of care at home, but expressed social challenges, such as the difficulty of identifying the main caregiver to ensure the continuation of care.

*Most caregivers, family members who are accompanying the patient, do participate in the planning. From the very first moment that some device is put in place, then we start with the guidelines. (E11-Nurse)*

*(...) they participate during hospitalization... especially with conscious patients, who are oriented, they have the right to choose, if they’re going to use the tube, if they want the tube (...) (E5 – Physician)*

*(...) it’s very important to identify this main caregiver... throughout hospitalization, but often this isn’t possible,(...) it depends on the family’s profile, social and cultural condition,(...) what they understand(...) (E10 – Social worker).*

In the practitioners’ view, when the team was able to provide the necessary information and develop self-care skills in the responsible discharge process, there was greater de-hospitalization and bed turnover and a lower rate of unnecessary re-hospitalization.

*(...) it was good for this, for us to prepare the companions, the caregivers... thus avoiding unnecessary re-hospitalization.(E2 – Physiotherapist)*

*(...) the team treatment, we can manage the patient more quickly, so we de-hospitalize, (...) we discharge more people, we generate less costs for the hospital, (...) (E5 – Physician)*

*Also, regarding the institution I see that maybe it helps financially, bed turnover...” (E8 – Nutritionist)*

However, they recognized deficiencies in the guidance provided to patients/family members/caregivers within the hospital, and a lack of follow-up after discharge. Only one doctor suggested telephone contact and home visits as strategies for assessing and clarifying doubts after discharge.

*(...) I think that sometimes there are still gaps, that we still see patients leaving without receiving any guidance (...) (E3-Speech-language therapist)*

*(...) it helps a lot, but I think it’s not 100% effective because of this lack of follow-up (...) (E7-Nutritionist)*

*(...) if there was more time, more days to give guidance, (...) because sometimes it’s all on the same day,.. the family is full of information and they get home and there’s doubt(...) to give guidance by video(...) not just in writing. (E3 – Speech-language therapist)*

*(...) making a brief telephone contact seven days after discharge, or/and making a home visit after discharge(...) could avoid a lot of readmissions(...) (E6 – Physician)*

Preparing the patient for post-hospitalization care requires effective communication between professionals and with the patient/family. According to the reports, the communication process during responsible discharge needs to be improved, both in terms of hospital structure and organization and in terms of dialogue and collaboration between people, services and the community.

### Interaction Between Professionals and Services for the Transition of Care

Interaction between professionals was facilitated by the use of the social media WhatsApp and by team meetings, even though it was still considered to be fragmented. There was recognition of the nurse as a mediator between the doctor and the interprofessional team and obstacles in receiving feedback from Primary Health Care.

*(...) WhatsApp groups make it much easier for [professionals] to work together. (E3 – Speech-language therapist)*

*(...) the nursing team (...) who make the link between the doctor and the multi-professional team (...)[nursing] is a key part of this, which helps a lot in this qualified discharge (...) (E8 – Nutritionist)*

*(...) there could be more team meetings (...) drawing up this plan together with all the professionals (...) it [team meetings] exists, but in a very fragmented way (...) (E10 – Social worker)*

*(...) we have multi-rounds twice a week, not every day (...) this could be improved (...) it’s still in the process of being improved (E2 – Physiotherapist)*

*(...) we don’t know if they’re doing [post-discharge follow-up] in the municipality because there’s no feedback, if they manage to do it (...) (E12 – Nurse)*

In addition to informal interaction, the team requires frequent interprofessional meetings and discussions to improve discharge planning. Despite the dialogical relationship, the collaborative construction of a common therapeutic project for transition and continuity of care is not routinely practiced by the team. In this context, a responsible discharge movement is identified, with weaknesses in the articulation of professional knowledge and the different points of the RAS.

### Workload Management for Better Transition and Continuity of Care

The professionals reported high demands for patients with criteria for inclusion in the responsible discharge process, as well as other activities, not allowing to attend all the team meetings and devote enough time to preparing the patient/family member for the transition and continuity of care. They also stated that some appointments were not made.

*(...) we have a very heavy workload, there are a lot of patients, so I can’t always take part in all the qualified discharge meetings, (...) sometimes the patient leaves and (...) we miss out, we can’t provide guidance (...) (E3 – Speech-language therapist)*

*(...) so that’s an average of thirteen patients a day (...) I think the time is always short (...) (E6 – Doctor)*

*(...) the time could be longer (...) as productivity is demanded; sometimes, we reduce the time (...) (E7 – Nutritionist)*

*(...) most of the time [the time] isn’t adequate, not least because of the demand, (...) it ends up being a time that varies a lot with the demand, the needs, the complexity of each patient. (E10 – Social worker)*

*(...) we always have two or three [patients] to deal with every day (...) there should be more time for us to dedicate to this (...) (E11 – Nurse)*

The overload of the clinical team hampers performance in the responsible discharge process, and was found in the investigated site, to be a critical factor in the transition and continuity of patient care.

## DISCUSSION

The responsible discharge process in the studied context was structured around interpersonal communication with significant deficiencies and centrality in medical decision-making. The findings highlight the importance of informational continuity^(11,12)^ and how challenging it is to empower the patient/family and develop self-care, as proposed in the theory of strengths^(15)^ and in national policy^(7)^. These difficulties were also emphasized in a recent study involving professionals from the Swiss health system^(16)^.

It is known that the exchange of information between team members is a determining factor in discharge planning and continuity of care^(20)^. Dialogue is one of the characteristics of the IPW^(2)^ and the definition of common, patient-centered objectives contributes to meeting the required needs^(16)^. However, the proposal of organizing services in order to meet the patient’s health demands in accordance with collaborative interprofessional practice^(2)^ confronts the persistent hegemonic model of care, especially in hospital institutions.

A Canadian study showed that medical collaboration is a differential in the discharge process. When physicians get involved with the other team members and engage personally and regularly in interprofessional discussions, a bond is built and there is a greater possibility of shared decisions with a positive impact on discharge planning^(21)^. This corroborates the findings of the present study. The interviewees valued the opportunities to meet and exchange knowledge between the different professionals, with special attention to the medical professional, and stressed the need to systematize and regularize team discussions. However, in practice, the definition of common objectives for patient discharge is not prioritized.

The different views on patient care, inherent in the training process; the difficulty in recognizing the role/knowledge of others; and the weakness of team communication^(22)^ negatively affect the process. There have been suggested educational interventions in order to develop more collaborative teams capable of understanding the different perspectives and reaching a consensus on the discharge plan^(16,20)^, and to strengthen relational continuity^(11,12)^, as well as informational continuity.

Interaction/communication between the professionals investigated was facilitated by the use of the social media WhatsApp. This technology has been widely used by health professionals to save time and improve sharing of information^(23)^; however, issues related to lack of regulation, confidentiality and data security have emerged^(24)^. Team meetings were also established, but work overload and a lack of systematization were negative aspects that led to fragmented care. More dialogical relationships require frequency, spaces and instruments to standardize the information transferred^(2)^.

Nurses have been recognized as mediators of interprofessional relationships and have been assigned the task of coordinating transition actions, i.e. management continuity^(4,5,11)^. Considering that this is a complex process that requires networking, time commitment and specific skills, European^(25,26)^ and North American^(27)^ institutions have adopted discharge management teams or nurses with exclusive dedication, that are called linkage, liaison, case managers or navigators, in order to coordinate and facilitate counter-referral and continuity of patient care. In these models, even if there is a process coordinator, there are mainly professionals who provide direct assistance to the patient/family in the hospital units.

In the Brazilian landscape, the exclusive role in discharge planning and continuity of care is still limited and, in most institutions, the actions are responsibility of the clinical team alongside other duties, which can compromise the effectiveness of the process^(28)^. The participants in this study reaffirmed this limitation, as they carried out various activities in the hospital routine, in addition to those related to responsible discharge, and the time constraints limited the quality and quantity of assessments and guidance they would need to carry out. This situation has also been identified by other authors^(8)^.

In addition to the informational, relational and managerial restrictions in the micro-context of practice, there was a lack of coordination between the different levels of care. Electronic medical records have been identified as a facilitating channel for the transfer of information between healthcare services^(16)^, but in addition to the interaction between different professionals, the decision-making process for hospital discharge involves inter – and extra-organizational factors^(8)^.

Only one of the interviewees (a physician) suggested monitoring the patient through telephone contact after discharge and scheduling a home visit depending on the care demands. It is known that telephone contact and home visits are important strategies for continuing and evaluating care after discharge^(5)^. Patients value this post-discharge follow-up experience^(29)^ and the combination of these interventions (nurse home visits and telephone follow-up) has proved effective in preventing unplanned readmissions^(13)^.

The interviewees confirmed the involvement of the patient/family/caregiver during hospitalization to clarify doubts about care and jointly decide on the proposed interventions. However, they revealed vulnerabilities in the process, such as: high workload, ineffective communication and social issues of the patient/family, which made the centrality and continuity of care unfeasible from the perspective of the patient’s autonomy.

The strengths portrayed by skills and personal and family resources, as well as the support network and services available^(15)^, were not clearly mentioned by the professionals who emphasized problems as the logic of care. In addition, the concentration of information on the last day of hospitalization, especially without prior planning and involvement, is a mistake in preparing for discharge. It is known that early identification of needs, participation in therapeutic decisions, guidance received, emotional support, as well as continuity of access, information and relationships are factors that have a significant impact on the patient’s/family member’s experience of the journey^(30)^. This experience of continuous care throughout the healthcare journey is the desirable process to achieve^(12)^.

The professionals interviewed associated better transfer of information and promotion of autonomy with better results (greater de-hospitalization and lower readmission rates). However, this effectiveness needs to be better investigated.

The hospital under study instituted the responsible discharge model more than seven years ago and the team has experience in the process, but in addition to piling up other activities added to the relevant tasks for the responsible discharge, interprofessional work and ensuring post-discharge care remain challenging. Structural and procedural issues, i.e., the organization of the service, collaboration between team members and the centrality of care for the patient still need to be improved, as well as better coordination within the RAS. It is necessary to invest in the triad composed by information, interpersonal relationships and coordination of care^(11)^ for better effectiveness in the transition and continuity of health care. Furthermore, the teams are challenged to expand care beyond the problems, understanding the individual and external strengths of the subject as potential for care after hospital discharge^(15)^.

As limitations, in this study, the reports represented the perception of some professionals from a teaching hospital at pandemic times. Other contexts, health situations or other professional categories could identify new demands. Individual interviews do not allow for interaction between professionals, which could have generated different contributions. Therefore, further research into responsible hospital discharge could explore both the professional experience, and the experience of patients, individually and/or collectively; and also investigate outcome indicators, confirming or refuting the views emerging from the group interviewed.

The findings of this research allow for providing guidance for health teams regarding the challenges to be overcome in the responsible hospital discharge process, helping managers as well, to plan, implement and evaluate critical points in the transition and continuity of patient care. Thinking of strengths-based care as a theoretical model guiding nursing practice and focused on the potential of the patient/family/community and the RAS, with an emphasis on collaborative practice, is another relevant aspect of this study. The dissemination of these attributes in the nurse training process and in educational actions in service are still fragile and need to advance. In addition, the model would need to go beyond nursing knowledge and interventions, in order to be recognized and adopted by the health team as a philosophy of comprehensive and continuous care that would apply to responsible discharge with transitions based on strengths.

Faced with still fragmented health care, professionals, managers, educators and researchers are provoked to (re)think effective strategies for collaborative interprofessional practices, centered on the patient and their strengths, with better articulation between the different points of the network to obtain successful experiences of continued care.

## CONCLUSION

The staff interviewed acknowledged that responsible hospital discharge can have a positive impact on both the institution (higher bed turnover) and the patient (reduction in unnecessary readmissions). However, interprofessional work needs to be strengthened to ensure the informational and relational continuity inherent in the process. The various activities undertaken by the professionals made it impossible to devote more time to responsible discharges, so effective management of the workload proved to be a relevant factor in improving the transition and continuity of patient care.
